# Zoonotic potential of *Enterocytozoon bieneusi* in pre-weaned Korean native calves

**DOI:** 10.1186/s13071-020-04175-2

**Published:** 2020-06-10

**Authors:** Sunwoo Hwang, Seung-Uk Shin, SuHee Kim, Ji-Hyoung Ryu, Kyoung-Seong Choi

**Affiliations:** 1 Department of Horse/Companion and Wild Animal Science, College of Ecology and Environmental Science, Kyungpook National University, Sangju, 37224 Republic of Korea; 2 Department of Animal Science and Biotechnology, College of Ecology and Environmental Science, Kyungpook National University, Sangju, 37224 Republic of Korea; 3 Gyeongsang National University Hospital, Jinju, 52727 Republic of Korea; 4 Foreign Animal Disease Division, Animal and Plant Quarantine Agency, Gimcheon, 39660 Republic of Korea

**Keywords:** *Enterocytozoon bieneusi*, Pre-weaned Korean native calf, Genotype, Zoonotic infection

## Abstract

**Background:**

*Enterocytozoon bieneusi* is the most common microsporidian species infecting humans and various animals worldwide. To date, there has been limited information on the prevalence of infection and genotypes of *E. bieneusi* in cattle in the Republic of Korea. Therefore, this study investigated the prevalence and genotypes of *E. bieneusi* circulating in pre-weaned Korean native calves and determined the age pattern of *E. bieneusi* infection and the relationship between *E. bieneusi* infection and diarrhea.

**Methods:**

The prevalence of *E. bieneusi* infection in pre-weaned Korean native calves was screened by polymerase chain reaction. PCR-positive products were sequenced to determine the genotype of *E. bieneusi*. A Chi-square analysis was used to compare the association between diarrhea and the infection rate of *E. bieneusi* in each age range or for all ages.

**Results:**

PCR and sequencing analysis revealed an overall prevalence (16.9%, 53/314) of *E. bieneusi* in pre-weaned calves. The prevalence of *E. bieneusi* was highest in September (36.2%), followed by March (28.3%). *Enterocytozoon bieneusi* infection was associated with diarrhea in calves (*χ*^2^ = 5.82, *P* = 0.016). Our results also indicated that *E. bieneusi* infection was significantly associated with calf age (*χ*^2^ = 11.61, *P* = 0.003), and the prevalence of *E. bieneusi* infection was significantly higher in calves aged 21–40 days-old (odds ratio: 2.90, 95% confidence interval: 1.54–5.45; *P* = 0.001) than in those aged 1–20 days-old. Interestingly, the association between *E. bieneusi* infection and diarrhea was observed only in calves aged 1–20 days-old (*χ*^2^ = 5.82, *P* = 0.010). We identified three known genotypes, BEB4 (*n* = 12), BEB8 (*n* = 21) and J (*n* = 16), and three novel genotypes, BEB8-like (*n* = 21), KCALF1 (*n* = 1) and KCALF2 (*n* = 1). The genotype BEB8 was the most prevalent among all age groups. All genotypes identified in this study exhibited zoonotic potential.

**Conclusions:**

To our knowledge, this is the first report of the genotype BEB4 in pre-weaned Korean native calves. Zoonotic *E. bieneusi* infection was prevalent in pre-weaned calves, indicating that cattle may play an important role as a reservoir host for *E. bieneusi* transmission to humans.
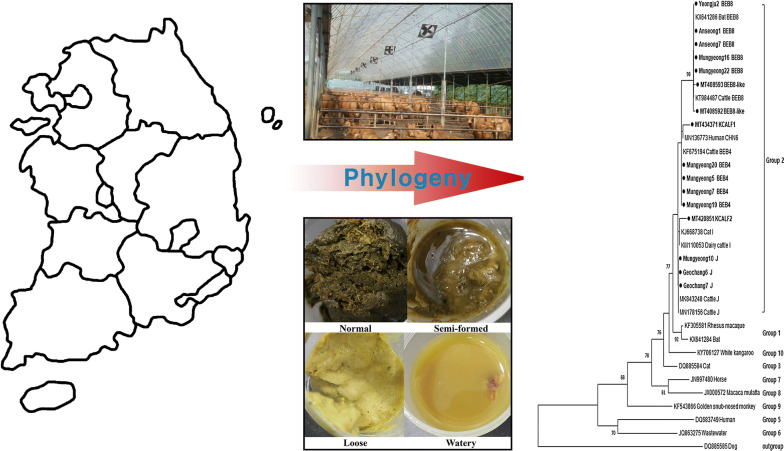

## Background

Microsporidia are obligate intracellular protozoan-like fungi that infect a wide range of invertebrates and vertebrates including humans [1]. Among approximately 17 human-pathogenic microsporidian species, *Enterocytozoon bieneusi* is the most common [2]. *Enterocytozoon bieneusi* usually causes gastrointestinal illnesses such as wasting syndrome and chronic diarrhea in the immunocompromised patients (AIDS or organ transplant recipients, patients with cancer); however, it also leads to asymptomatic and symptomatic infections in immunocompetent individuals [3–7]. This species is primarily transmitted through the fecal-oral route, and *E. bieneusi* spores from seemingly healthy animals, humans, and contaminated water or food could be potential sources of infection [8]. Despite the clinical and public health importance of *E. bieneusi*, the implication has not been emphasized because of the low incidence rate in most of the countries.

Genotyping of *E. bieneusi* and assessment of its host specificity and zoonotic potential are dependent on the sequence analysis of the ribosomal internal transcribed spacer (ITS) [9, 10]. Currently, 474 *E. bieneusi* genotypes have been identified in various hosts. In a phylogenetic analysis, the genotypes of *E. bieneusi* have been clustered into at least 11 groups (Groups 1–11) [11]. Group 1 contains the most genotypes found in humans and is considered to be zoonotic. Groups 2–11 have also been found in humans and are associated with various hosts (ruminants, non-human primates, horses, dogs, rabbits, bats, pigs, meerkats, bears, alpacas, chickens and pigeons) [11] and wastewater [12, 13]. To date, more than 50 *E. bieneusi* genotypes have been identified in cattle, most of which belong to Group 2 [14]. Among them, some genotypes (BEB4, BEB6, I and J) were detected in humans [15–18], suggesting that cattle can serve as potential reservoirs of human infection.

According to several studies, BEB4, I and J are common genotypes of *E. bieneusi* found in pre-weaned calves worldwide [17, 19–21]. However, there is limited information available about the infection rate and genotype distribution of *E. bieneusi* in pre-weaned Korean native calves. Therefore, this study sought to investigate the prevalence and genotypes of *E. bieneusi* circulating among pre-weaned Korean native calves, the age pattern of *E. bieneusi* infection, and the relationship between *E. bieneusi* and diarrhea.

## Methods

### Sample collection

From January to October 2018, a total of 314 fecal samples were collected directly by an experienced veterinarian from the rectum of pre-weaned Korean native calves (aged ≤ 60 days-old) on 10 different farms in the Republic of Korea (ROK), transported to the Animal Immunology Laboratory of Kyungpook National University, ROK, in a cooler with ice packs, and stored at 4 °C before DNA extraction. The fecal consistency of each calf was categorized as normal or diarrheic according to its physical characteristics.

### DNA extraction and PCR amplification

Genomic DNA was extracted using the QIAamp Fast DNA Stool Mini Kit (Qiagen, Hilden, Germany) using approximately 200 mg of each fecal sample according to the manufacturer’s instructions and then stored at – 20 °C until used in PCR analysis. *Enterocytozoon bieneusi* was screened based on the ITS region of the rRNA by nested PCR under the following conditions: 94 °C for 3 min; followed by 35 cycles of 94 °C for 45 s, 55 °C for 45 s and 72 °C for 1 min; and a final extension step at 72 °C for 10 min [22]. The amplified fragment was ~ 390 bp. *Cryptosporidium parvum* and *Giardia duodenalis* infections were also detected using the 60 kDa glycoprotein (*gp60*) and β-giardin genes [23–25]. Secondary PCR products were separated by electrophoresis on 1.5% agarose gels and then visualized after staining with ethidium bromide. In this study, only samples showing a good sequencing result were considered to be positive for *E. bieneusi*.

### Sequencing and phylogenetic analysis

The secondary PCR products were purified using the AccuPower PCR Purification Kit (Bioneer, Daejeon, ROK) and used for direct sequencing (Macrogen, Daejeon, ROK). In order to determine the genotype of *E. bieneusi*, the nucleotide sequences obtained in this study were aligned using ClustalX and were compared with the reference sequences from the GenBank database. A phylogenetic tree was constructed based on the nucleotide alignments using the maximum-likelihood method implemented in the MEGA 7 software [26] and bootstrap analysis was used to evaluate the robustness with 1000 replicates.

### Statistical analysis

Statistical analysis was performed using SPSS Statistics 25 software package for Windows (SPSS Inc., Chicago, IL, USA). Chi-square test was used to compare the association between diarrhea and the infection rate of *E. bieneusi* in each age range or all of the ages investigated in this study. In addition, the prevalence of *E. bieneusi* for each age range was determined using binary univariate logistic regression models. The odds ratio (OR) and 95% confidence intervals (CI) were calculated to determine the likelihood of association. A *P-*value of ≤ 0.05 was considered to be statistically significant.

## Results

### Prevalence of *E. bieneusi*

The overall prevalence of *E. bieneusi* was found to be 16.9% (53/314) in pre-weaned Korean native calves regardless of diarrhea. Among the 10 different farms examined, *E. bieneusi* was detected in 6 farms (Table 1). We compared the infection rate of *E. bieneusi* according to the month of sampling. As shown in Table 2, the prevalence of *E. bieneusi* was highest in September (36.2%), followed by March (28.3%) and October (14.7%); however, *E. bieneusi* infection was not detected in July and August. When *E. bieneusi* infection was compared according to the fecal consistency, there were 11.9% and 22.1% infection rates in both diarrheic and normal feces, respectively. Co-infection with *E. bieneusi* and *C. parvum* was not detected; however, co-infection with *E. bieneusi* and *G. duodenalis* was observed in diarrheic (6.3%, 10/314) and normal feces (1.9%, 3/314). Although there was no statistically significant difference, the risk of diarrhea was increased by 3.36-fold during co-infection with *E. bieneusi* and *G. duodenalis* (95% CI: 0.91–12.43, *P* = 0.056; Table 3). *Enterocytozoon bieneusi* infection was associated with diarrhea (*χ*^2^= 5.82, *df* = 1, *P* = 0.016; Table 4). *Enterocytozoon bieneusi-*positive samples were compared according to the age group of the calves. As shown in Table 4, the prevalence of *E. bieneusi* was the highest in calves aged 21–40 days-old, followed by those aged 41–60 days-old and 1–20 days-old (*χ*^2^ = 11.61, *df* = 2, *P* = 0.003). The risk of being positive to *E. bieneusi* was 2.9-fold higher in calves aged 21–40 days-old (95% CI: 1.54–5.45, *P* = 0.001) than in those aged 1–20 days-old. The association between *E. bieneusi* infection and diarrhea according to the age group was analyzed by the Chi-square test. *Enterocytozoon bieneusi* infection was found to be associated with diarrhea only in calves aged 1–20 days-old (*χ*^2^ = 6.61, *df* = 1, *P* = 0.010; Table 5).

**Table 1:** Prevalence and genotypes of *E. bieneusi* identified in pre-weaned Korean native calves

Region	*N*	No. of positive samples	ITS genotype
Anseong	39	4	BEB8 (*n* = 2), J (*n* = 2)
Geochang	78	22	BEB4 (*n* = 3), BEB8 (*n* = 7), BEB8-like^a^ (*n* = 1), J (*n* = 11)
Gimje	71	1	KCALF2^a^ (*n* = 1)
Gyeongju	6	0	–
Jeongeup	1	0	–
Mungyeong	82	21	BEB4 (*n* = 9), BEB8 (*n* = 8), BEB8-lik^a^ (*n* = 1), J (*n* = 2), KCALF1^a^ (*n* = 1)
Naju	1	0	–
Sangju	2	1	J (*n* = 1)
Yechoen	1	0	–
Youngju	33	4	BEB8 (*n* = 4)
Total	314	53	BEB4 (*n* = 12), BEB8 (*n* = 21), BEB8-like^a^ (*n* = 2), J (*n* = 16), KCALF1^a^ (*n* = 1), KCALF2^a^ (*n* = 1)

^a^Novel genotypes found in this study

*Abbreviation*: *N*, number of calves examined

**Table 2:** Prevalence of *E. bieneusi* in pre-weaned Korean native calves according to the month

Month	No. positive/No. examined	Prevalence (%)
January	0/2	0
March	15/53	28.3
April	6/68	8.8
May	3/27	11.1
June	1/23	4.3
July	0/12	0
August	0/7	0
September	17/47	36.2
October	11/75	14.7
Total	53/314	16.9

**Table 3:** Detection rates of all pathogen species in pre-weaned Korean native calves according to the diarrhea status

Pathogen	Positive in diarrhea samples (*n* = 160)	Positive in non-diarrhea samples (*n* = 154)	*χ*^2^ (*P-*value)	OR (95% CI)
*Cryptosporidium parvum*	11 (6.9%)	14 (9.1%)	0.53 (0.468)	0.74 (0.32–1.68)
*Giardia duodenalis*	23 (14.4%)	18 (11.7%)	0.50 (0.480)	1.27 (0.66–2.46)
*Enterocytozoon bieneusi*	19 (11.9%)	34 (22.1%)	5.82 (0.016)*	0.48 (0.26–0.88)
*E. bieneusi* + *G. duodenalis*	10 (6.3%)	3 (1.9%)	3.66 (0.056)	3.36 (0.91–12.43)

*P < 0.05

**Table 4:** Association between physical variables and *E. bieneusi* infection in pre-weaned Korean native calves

Variable	Frequency of *E. bieneusi* positivity (%)	*χ*^2^ (*P* value)	OR (95% CI)
Fecal consistency			
Non-diarrhea (Ref.)	34/154 (22.1)	5.82 (0.016)*	1.00
Diarrhea	19/160 (11.9)		0.48 (0.26–0.88)*
Age (days)			
1–20 (Ref.)	19/173 (11.0)	11.61 (0.003)**	1.00
21–40	30/114 (26.3)		2.90 (1.54–5.45)**
41–60	4/17 (14.8)		1.41 (0.44–4.51)

*P < 0.05, **P < 0.005 *vs* reference (Ref.)

**Table 5:** Association between diarrhea and presence of *E. bieneusi* in pre-weaned Korean native calves according to age

Age (days)	Fecal consistency	Frequency of *E. bieneusi* positivity (%)	*χ*^2^ (*P-*value)	OR	95% CI
1–20	Diarrhea	6/102 (5.9)	6.61 (0.010)*	0.28	0.10–0.77
	Non-diarrhea	13/71 (18.3)			
21–40	Diarrhea	10/44 (22.7)	0.48 (0.490)	0.74	0.31–1.76
	Non-diarrhea	20/70 (28.6)			
41–60	Diarrhea	3/14 (21.4)	1.01 (0.596)	3.27	0.30–36.31
	Non-diarrhea	1/13 (7.7)			

*P < 0.05 *vs* reference (Ref.)

### Genotypes of *E. bieneusi*

**Fig. 1:**
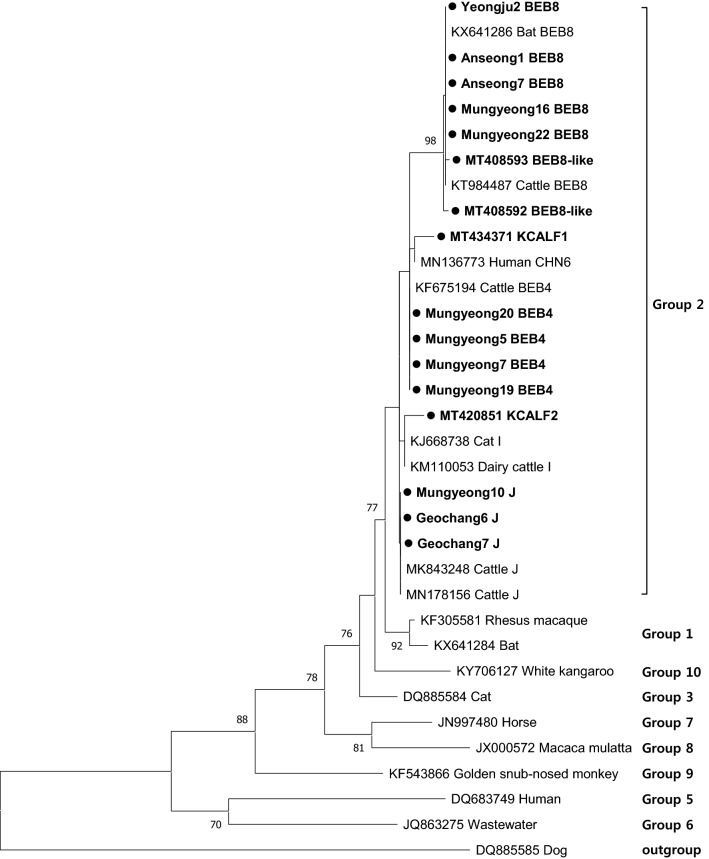
Phylogenetic relationships of *Enterocytozoon bieneusi* genotypes identified in this study and other reported genotypes based on the 243 bp internal transcribed spacer gene sequences. The tree was constructed using MEGA 7 software with the maximum-likelihood method. Numbers at the nodes of the tree indicate bootstrap values as a percentage of 1000 replicates that support each phylogenetic branch. The isolates identified in this study are marked in bold type with a circle symbol

To determine the genotypes of *E. bieneusi* detected in pre-weaned Korean native calves, a total of 53 ITS-positive samples were sequenced. The length of a 243 bp sequence was used to construct the phylogenetic tree. Three distinct genotypes, BEB4 (*n* = 12, identical to GenBank: KF675194), BEB8 (*n* = 21, identical to GenBank: KT984487) and J (*n* = 16, identical to GenBank: MN178156) were found. Moreover, three novel genotypes, BEB8-like (*n* = 2), KCALF1 (*n* = 1) and KCALF2 (*n* = 1), were identified. Of the 53 ITS-positive samples, 16 sequences were included in the phylogenetic tree, and all of the genotypes identified in this study belonged to the zoonotic Group 2 (Fig. 1). Among them, BEB8 was found to be the most prevalent genotype in pre-weaned calves regardless of diarrhea. In contrast, the genotypes KCALF1 and KCALF2 were found only in one calf aged 16 days and 10 days with diarrhea, respectively. In particular, the genotypes BEB8 and J were detected in all age groups (Table 6). BEB4 was found only in calves aged up to 40 days-old and only on two farms. Calves aged 1–20 days-old had a higher diversity of genotypes, with six genotypes being found and the diversity of genotypes decreased with age (Table 6). The BEB8-like genotype showed only one nucleotide difference from the genotype BEB8. The novel genotype KCALF1 differed by four nucleotides relative to CHN6 (GenBank: MN136773), which was found in the feces of humans in China. KCALF2 also showed four nucleotide differences from genotype I (GenBank: MT231513) isolated from cattle feces. As shown in Table 1, the distribution of *E. bieneusi* genotypes on the farms was different. Three farms (Gimje, Sangju and Yeongju) had only one genotype, whereas the others had two to five genotypes (Table 1). To the best of our knowledge, this is the first report of the presence of the genotype BEB4 in pre-weaned Korean native calves.

**Table 6:** Genotype distribution of *E. bieneusi* according to age in pre-weaned Korean native calves

Age (days)	BEB4	BEB8	BEB8-like	J	KCALF1	KCALF2	Total
1–20	4	7	1	5	1	1	22
21–40	8	11	1	10	–	–	30
41–60	–	3	–	1	–	–	4
Total	12	21	2	16	1	1	53

## Discussion

The present study showed that the infection rate of *E. bieneusi* in pre-weaned Korean native calves was 16.9%, which is similar to that reported in other studies for cattle in the ROK and several other countries [17, 27–30]. The prevalence of *E. bieneusi* in pre-weaned calves varied from 0% to 50% depending on the farm. This variation could be attributed to nutrition, herd management practices, health of the animal, and hygiene. Interestingly, *E. bieneusi* infection appears to be closely related to seasonal differences. According to our results, *E. bieneusi* infection tended to occur primarily in September (36.2%) and March (28.3%); in contrast, the incidence rate of *E. bieneusi* was rather low in warmer seasons with no infections in July and August. This result is different from that of a previous study reporting a higher prevalence during warmer seasons in the ROK [27]. The differences between these two studies might be explained by the difference in the number of samples collected each month and the age of the calf. However, our results were fairly consistent with those of a study conducted in China, which reported a higher prevalence in spring [31]. Although the results are inconclusive, the transmission of *E. bieneusi* may be related to seasonal variations. Thus, further studies are required to investigate the association between *E. bieneusi* infection and seasonal variations.

In this study, according to the Chi-square analysis, *E. bieneusi* infection was associated with diarrhea; however, the infection rate was not high in diarrheic feces. Although *E. bieneusi* was detected in diarrheic feces, it is unlikely that *E. bieneusi* is associated with diarrhea in pre-weaned calves (OR = 0.48, 95% CI: 0.26–0.88, *P* = 0.016; Table 3). However, a previous study performed in China revealed that *E. bieneusi* infection increased 2.5-fold in pre-weaned calves with diarrhea (95% CI: 1.7–3.8, *P* ≤ 0.0001) compared with those without diarrhea, and most of all, *E. bieneusi* infection was associated with diarrhea [32]. The difference between the two groups could be explained by the number of samples and the age of the calf. Thus, the relationship between *E. bieneusi* infection and diarrhea should be determined through further investigation.

Interestingly, among the three pathogens examined, the infection rate of *E. bieneusi* was the highest in pre-weaned Korean native calves. This might have been overlooked in diagnostic tests due to uncertainty regarding the role of *E. bieneusi* as a pathogen in calf diarrhea. Furthermore, it is possible that the significance of *E. bieneusi* has not been prominently recognized in the field. Results of the present study showed that co-infection with *E. bieneusi* and *G. duodenalis* was not statistically significant (*P* = 0.056; Table 3); however, it was 3.36-fold more likely to cause diarrhea (95% CI: 0.91–12.43) compared with a single infection with *E. bieneusi* in calves. Our result was inconsistent with that reported in China; co-infection with *E. bieneusi* and *G. duodenalis* was significantly associated with diarrhea [32]. In this study, the number of positive samples co-infected with two pathogens was small; thus, these results failed to demonstrate an association between diarrhea and co-infection in pre-weaned calves. Although it remains unclear, such co-infection might increase the severity and duration of diarrhea in calves. More epidemiological investigations are required to determine whether the occurrence of diarrhea is more common in calves co-infected with *E. bieneusi* and *G. duodenalis*.

The prevalence of *E. bieneusi* in calves was significantly associated with the age of the calf. The infection rate of *E. bieneusi* was the highest in calves aged 21–40 days-old, followed by calves aged 41–60 days-old, and 1–20 days-old. In comparison with calves aged ≤ 20 days-old, the risk of *E. bieneusi* infection was 2.9-fold higher in calves aged 21–40 days-old (*P* = 0.003; Table 4). A possible explanation is that the immune status of calves in this age group may result in a higher susceptibility to *E. bieneusi* infection due to the loss of the maternal antibodies [33]. To date, several studies have demonstrated age-related prevalence patterns of *E. bieneusi* infection [19, 28, 29, 34]. However, in contrast to our findings, the prevalence of *E. bieneusi* has been found to increase with age [19, 35, 36]. In the ROK, there are limited studies on *E. bieneusi* infection in cattle; thus, it is not possible to compare the prevalence of *E. bieneusi* according to age group. In addition, there is no information on the transmission route of *E. bieneusi* on the farms examined; however, *E. bieneusi* infection may be related to the hygiene status of farms rather than the calf age. Therefore, to prevent *E. bieneusi* infection, the farming management system should be improved, which could include no contact with contaminated food and water, cleaning, and disinfection.

We also investigated the association between *E. bieneusi* infection and diarrhea according to the age group. Our results revealed that there was a significant correlation between *E. bieneusi* infection and diarrhea in calves aged 1–20 days-old (*P* = 0.010; Table 5). However, this has been shown to be associated with a low incidence of diarrhea in *E. bieneusi*-infected calves. Based on the results, it is unlikely that *E. bieneusi* is the primary pathogen that causes diarrhea in pre-weaned Korean native calves. Cattle may be a source of environmental contamination by *E. bieneusi*. Therefore, *E. bieneusi* infection in calves should be considered as a zoonotic potential rather than a causative agent of diarrhea.

In the present study, sequence analysis of the ITS gene from 53 *E. bieneusi*-positive isolates identified six genotypes (BEB4, BEB8, J, BEB8-like, KCALF1 and KCALF2) belonging to zoonotic Group 2. Unlike previous studies, the genotype BEB8 was the most prevalent in pre-weaned Korean native calves and was found in 45.3% (24/53) of the positive samples. Moreover, this genotype was commonly identified in all age groups regardless of diarrhea. Several studies have reported that the genotype BEB8 can be found not only in cattle [29, 37, 38] but also in bats [39] and rabbits [40], indicating that this genotype might have a potential risk for zoonotic infection in humans. Genotype J, identified in 30.2% (16/53) of *E. bieneusi*-positive samples, was the second most common genotype in all age groups. BEB4 was the third most common genotype and found in calves only up to 40 days-old. BEB4 has been identified as a zoonotic genotype in cattle in many countries; however, it was first detected in the ROK. Interestingly, genotype I, with a wide range of hosts, was not detected in this study. In contrast, a previous study conducted in the ROK reported the presence of genotype I in three cattle [26]. This can be attributed to the low incidence of genotype I in cattle in the ROK compared to that of other countries. The present study reported the identification of three novel genotypes in pre-weaned Korean native calves, indicating that high genetic diversity exists in the *E. bieneusi* ITS region. Furthermore, the genotypes BEB4, BEB8 and J were common genotypes in pre-weaned Korean native calves. The differences in the distribution of *E. bieneusi* in pre-weaned calves according to farms may be attributed to the geographical location and the farm management system. All genotypes identified in this study have a possible zoonotic potential, suggesting that cattle play an important role as a reservoir host in *E. bieneusi* transmission to humans.

## Conclusions

This study evaluated the presence and genotypes of *E. bieneusi* detected in pre-weaned Korean native calves. *Enterocytozoon bieneusi* infection was associated with diarrhea in calves aged 1–20 days-old, and the prevalence of *E. bieneusi* was significantly higher in calves aged 21–40 days-old. ITS sequencing identified six genotypes (BEB4, BEB8, J, BEB8-like, KCALF1 and KCALF2), with BEB8 being the most prevalent genotype in pre-weaned Korean native calves. The identification of zoonotic genotypes in pre-weaned calves suggests that these animals could play an important role as reservoir hosts for zoonotic infections.

## Data Availability

All data generated or analyzed during this study are included in the article. The nucleotide sequences obtained in the present study have been deposited in the GenBank database under the accession numbers MT408592-MT408593 for genotype BEB8-like, MT434371 for genotype KCALF1, and MT420851 for genotype KCALF2.

## Abbreviations

CI: confidence interval
ITS: internal transcribed spacer
OR: odds ratio
PCR: polymerase chain reaction
ROK: Republic of Korea
